# Air Pollution and Serum Glucose Levels: A Population-Based Study

**DOI:** 10.1097/MD.0000000000001093

**Published:** 2015-07-13

**Authors:** Maayan Yitshak Sade, Itai Kloog, Idit F. Liberty, Itzhak Katra, Lena Novack, Victor Novack

**Affiliations:** From the Clinical Research Center, Soroka University Medical Center, Faculty of Health Sciences, Ben-Gurion University of the Negev, Be’er-Sheva, Israel (MYS, VN); Department of Geography and Environmental Development, Faculty of Humanities and Social Sciences, Ben-Gurion University of the Negev, Be’er-Sheva, Israel (IK, IK); Department of Internal Medicine, Soroka University Medical Center, Be’er-Sheva, Israel (IFL); and Department of Public Health, Faculty of Health Sciences, Ben-Gurion University of the Negev, Be’er-Sheva, Israel (LN)

## Abstract

Recent studies demonstrated an adverse effect of chronic exposure to air pollution (AP) on metabolic syndrome and its components. In a population-based study, we investigated the association between exposure to ambient AP and serum glucose (SG), among subjects with normal glucose, impaired fasting glucose (IFG), and diabetes mellitus (DM).

We included 1,063,887 SG tests performed in 131,882 subjects (years 2001–2012). Exposure data included daily levels of SO_2_, NO_2_ and other pollutants of industrial, traffic, and nonanthropogenic sources. Demographical, clinical, and medications purchase data were assessed. Log-transformed SG levels were analyzed by linear mixed models adjusted for seasonal variables and personal characteristics.

SG increases (%increase [95% CI]), among subjects with normal glucose, IFG, and DM, respectively, were associated with 6.36 ppb increase of NO_2_ measured 24 to 72 hours before the test (0.40% [0.31%; 0.50%], 0.56% [0.40%; 0.71%], and 1.08% [0.86%; 1.29%]); and with 1.17 ppb increase of SO_2_ measured 24 hours before the test (0.29% [0.22%; 0.36%], 0.20% [0.10%; 0.31%], and 0.33% [0.14%; 0.52%]). Among DM population, weakest association was observed among patients treated with Metformin (0.56% increase in SG [0.18%; 0.95%]).

In conclusion, NO_2_ and SO_2_ exposure is associated with small but significantly increased levels of SG. Although DM patients were found to be more susceptible to the AP induced SG variations, Metformin treatment seem to have a protective effect. Given the chronic lifetime exposure to AP and the broad coverage of the population, even small associations such as those found in our study can be associated with detrimental health effects and may have profound public health implications.

## INTRODUCTION

Recent studies demonstrated an adverse effect of long-term exposure to air pollution (AP) on metabolic syndrome and its components, such as diabetes mellitus (DM) and hypertension,^1–3^ adding risk to known factors such as lifestyle, obesity, and inactivity.^4^

The data on the effect of the specific pollutants on glucose homeostasis—a major component of the metabolic syndrome—are scarce. One study found positive association of fasting serum glucose (SG) only with ozone, and no other pollutants.^5^ Others found positive significant associations between fasting SG levels, insulin resistance, and particular matter smaller than 10 μm in diameter (PM_10_),^6^ particular matter smaller than 2.5 μm in diameter (PM_2.5_),^7,8^ sulfur dioxide (SO_2_),^6^ and nitrogen dioxide (NO_2_).^9–11^ Studies suggested that the pathophysiology explaining a possible association between AP and glucose levels is linked to an inflammatory response which may disrupt the lipid and glucose metabolism process.^3,12,13^

Current evidence is highly diverse in terms of exposure assessment, outcome definitions, and the estimates of association found. The association between AP exposure and glucose homeostasis can be confounded by time factors,^5^ seasonality factors,^5,7^ and personal characteristics: age,^3,5,7,14,15^ gender,^3,5,15^ ethnicity,^7,15^ comorbidities,^3^ socioeconomic status (SES),^3,5,15^ and other confounders.^3^

Trying to address the potential biases, we performed a population-based analysis of over 1 million tests, collected over 12 years in Southern Israel (Negev). We aimed to explore the association between AP (PM_10_, carbon monoxide (CO), SO_2_, NO_2_, and O_3_) and SG and glycosylated hemoglobin (HbA1c) variations, and to assess the potential interactions by the presence of diabetes and impaired glucose tolerance and type on antidiabetic treatment.

This research was supported by grant award No. SGA 1303 from the environment and health fund, Israel.

## METHODS

### Study Population

We included all fasting SG tests of Clalit Health Services (CHS), Health Maintenance Organization (HMO) members, performed in southern Israel between the years 2001 and 2012. Health data were retrieved from CHS database. CHS is the largest HMO in Israel; it insures approximately 70% of the Negev population of 730,000 residents.^16^ All blood tests of CHS members in the Negev are analyzed by a single laboratory. The demographic, clinical, laboratory and medication prescription data of CHS members are fully computerized and are available at the patient level. We obtained the following patient data: age, gender, ethnicity, comorbidities, medications, and SES. The latter was stratified into 3 levels: low, intermediate, and high. SES was assigned based on the SES of the population residing in the close proximity to the subjects’ primary clinic, according to the definitions of the central bureau of statistics (CBS). Ethnicity was assigned for each city or settlement of residence by the CBS and was determined according to the religion of the majority of the population residing in it.^17^

We excluded children (under 18 years of age), and those residing further than 20 km from the available monitoring stations. The population excluded was similar to the subjects represented in the study in terms of age, gender, and ethnicity distribution.

### Clinical Definitions

We defined the patient DM or impaired fasting glucose (IFG) status in accordance to the American Diabetes Association (ADA) criteria.^18^*Diabetes diagnosis* was established if one of the following was present: documented physician confirmed diagnosis, antidiabetic medication purchase, more than 1 measurement of fasting SG equal or higher than 126 mg/dL or more than 1 measurement of HbA1c ≥6.5%. Patients were defined as having *IFG* if case of a record of more than 1 fasting SG between 100 and 125 mg/dL, or more than 1 measurement of HbA1c between 5.7% and 6.5%, or only 1 fasting SG level higher than 125 mg/dL, or only 1 HbA1c ≥6.5% in the presence of at least 1 additional fasting SG level between 100 and 125 mg/dL and at least 1 HbA1c level between 5.7% and 6.5%.^18^ On an event of multiple tests available per a patient the most severe status of the disease was assigned, that is, patients meeting DM criteria once during the study period, were considered as such through the entire follow-up time.

Tests performed during hospitalizations were excluded from the analysis.

### Air Pollution and Meteorological Data

Daily data on air pollutants and meteorological variables (air temperature and relative humidity) for the period of 2001 to 2012 were obtained from the monitoring site located in the center of the largest city (Beer-Sheva) in the Negev area. This monitoring station is simultaneously recording data (every 5 minutes) of the following pollutants: PM_10_, CO, SO_2_, NO_2_, and O_3_. Pollutants values higher than the 98th percentile were defined as outliers and were imputed with the value of the 98th percentile.

Blood tests are performed between 7:00 and 10:00 am in all primary clinics in southern Israel. Since the exact time of the test was not available, we used the calculated averages of concentrations of the pollutants over 24 hours (from 10:00 in the previous day to 10:00 am in the day of the test) as well as the temperature and relative humidity—24, 48, and 72 hours before the day of the test.

### Statistical Analysis

Results are presented by mean ± SD, interquartile range (IQR) and range for continuous variables and as percentages for categorical data.

Coefficient of variation (CV) of SG was calculated for each subject. Low and high variability of SG results were defined according to CV values lower than the 10th percentile, and higher than the 90th percentile, respectively.

Analyses were performed separately among subjects with DM, IFG, and normal glucose. Log-transformed SG levels were modeled by mixed linear models, accounting for repeated tests within each subject. We used multipollutant models, inclusive of 24, 48, or 72 hours averages of pollutants. Models were adjusted for average value of temperature and relative humidity, day of the week, year, age, gender, hypertension, ethnicity, and SES. Models for DM patients were also adjusted for the purchase of anti diabetic drugs during the 3 preceding months. Coefficients were antilog transformed to the original units, and results are presented as percent change in SG levels and 95% confidence intervals (CI).

In a subgroup analysis among the DM patients, we stratified by the type of treatment: no medications, Insulin, Metformin, or other antidiabetic drugs (GLP1 agonists, DPP4 inhibitor, alpha glucosidase inhibitor, sulphonylurea, meglitinides, and thiazolidinediones).

The log-transformed HbA1c levels were modeled as well. Since HbA1c levels represent the mean SG levels over approximately 3 months, exposure to pollutants was assigned as a 3 months moving average of the pollutants concentrations.

### Sensitivity Analysis

SES data were available for 87% of the study population. We performed sensitivity analyses to verify our results with the imputation of median and maximal SES levels.

Analyses were performed in SAS 9.4 (SAS Institute, Inc., Cary, NC) and R3.1.0 software.

The study has been approved by the institutional review board of Soroka University Medical Center (SUMC).

## RESULTS

### Population

**FIGURE 1:**
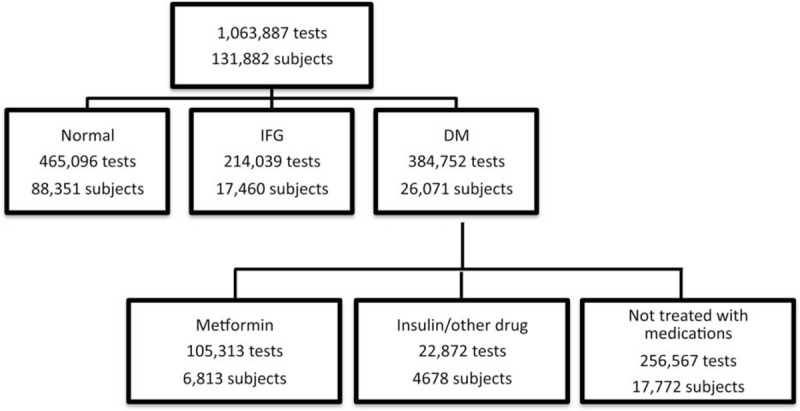
The total number of tests and subjects included in the study, by 3 comparison groups: subjects with normal glucose, impaired fasting glucose (IFG), and diabetes mellitus (DM). DM patients are stratified by the treatment type.

We identified 1,063,887 SG tests of 131,882 region residents eligible for the study (Figure 1). DM patients (26,071 subjects) were 63 years old on average, 68% were treated with anti diabetic drugs. Subjects (105,811 subjects) with no evidence of DM or IFG were younger, and 2.22% (1961 subjects) were diagnosed with ischemic heart disease and/or myocardial infarction (Table 1).

**TABLE 1:**
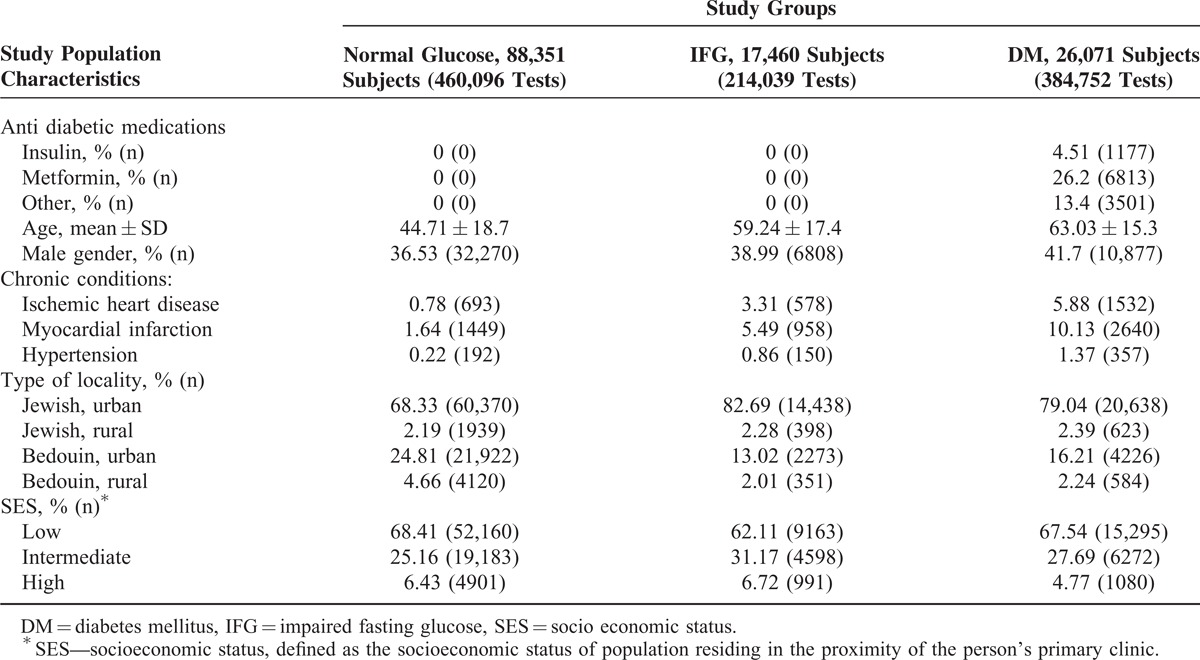
Study Population Characteristics

### Meteorological and Pollution Data

Table 2 shows summary statistics of AP and meteorology data during the study period. Air pollutants IQR during the study period were as follows: CO 0.19 ppm, NO_2_ 6.36 ppb, SO_2_ 1.17 ppb, O_3_ 13.79 ppb, and PM_10_ 26.44 μg/m^3^. The air temperatures are relatively high most of the year. The IQR of the 24-hour mean air temperature ranged between 14.4 and 24.9 °C reaching maximal mean air temperature of 33.7 °C (Table 2).

**TABLE 2:**
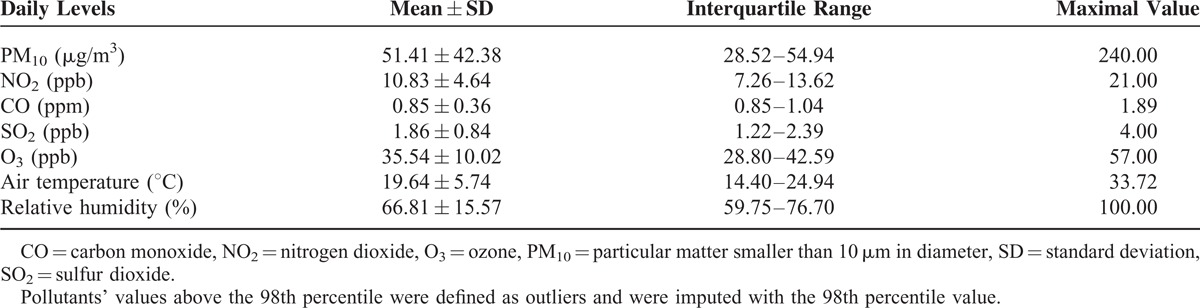
Summary Statistics for Daily 24 hours Average Air Pollutant and Meteorological Data (2001–2012)

### Estimating the Effect of Air Pollutants on SG and HbA1c

Linear mixed models were employed to estimate the association between AP exposure and SG levels and HbA1c in all study groups. AP and meteorological factors were not highly correlated in our data, with a highest correlation estimate observed for NO_2_ and SO_2_ (r = 0.34, *P* < 0.01).

### NO_2_

In all groups, SG levels were positively associated with IQR elevations of average NO_2_ concentrations 48 and 72 hours before the blood test. Stronger associations were observed with NO_2_ concentrations 72 hours before the blood test. Association observed among subjects with DM were stronger: 1.08% increase (95% CI: 0.86%; 1.29%), compared to 0.40% increase (95% CI: 0.31%; 0.50%) among subjects with normal glucose levels; and 0.56% increase (95% CI: 0.40%; 0.71%) among subjects with IFG.

### SO_2_

In all groups, SG increases were more pronounced when assessing exposure to SO_2_ concentrations 24 hours before the blood test, compared to prolonged periods of exposure. The highest increase in SG was observed among subjects with DM: 0.33% increase (95% CI: 0.14%; 0.52%), compared to 0.29% increase in SG (95% CI: 0.22–0.36%) among subjects with normal glucose levels; and 0.20% increase in SG (95% CI: 0.10–0.31%) among subjects with IFG (Table 3).

**TABLE 3:**
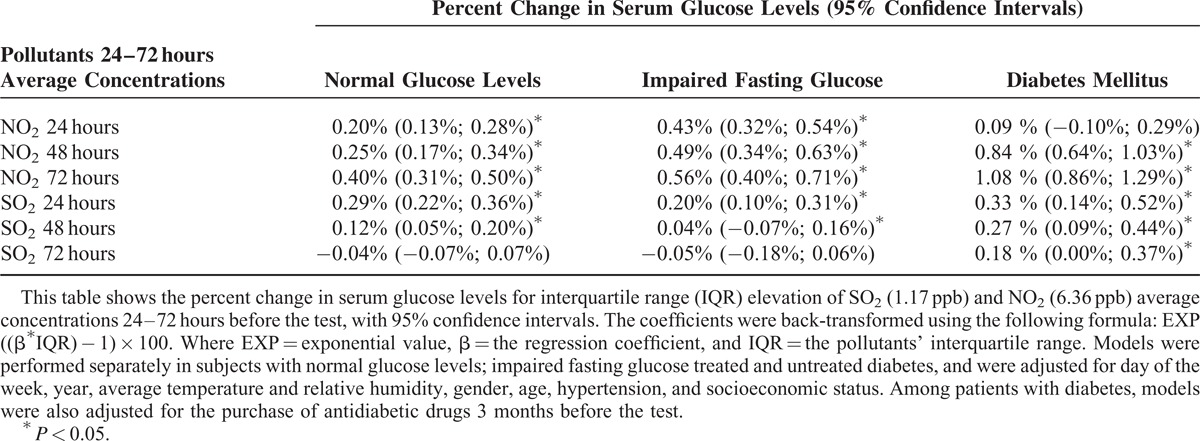
The Percent Change in Serum Glucose Levels, Associated With NO_2_ and SO_2_ Concentrations, Among Subjects With Normal Glucose Levels; Impaired Fasting Glucose and Diabetes Patients

No associations were found with CO, PM_10_, or O_3_ average concentrations 24 to 72 hours before the test, in all study subgroups.

### Identification of Susceptible Population

**FIGURE 2:**
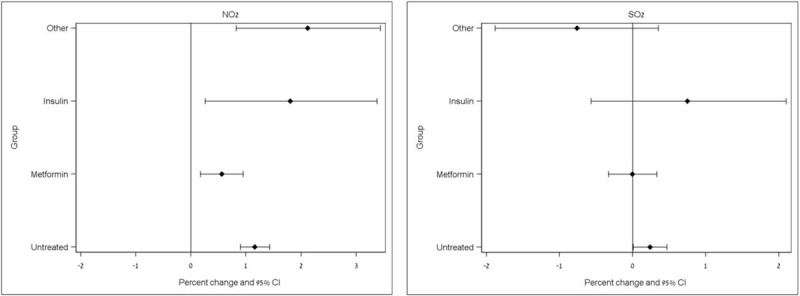
The percent change in serum glucose levels for IQR elevation of NO_2_ (6.36 ppb) and SO_2_ (1.17 ppb) concentration 72 hours before the test, with 95% confidence intervals. The coefficients were back-transformed using the following formula: EXP ((β^∗^IQR) − 1)×100. Where EXP = exponential value, β = the regression coefficient, and IQR = the pollutants’ interquartile range. Models were performed separately among untreated patients with diabetes, and among patients treated with Insulin, Metformin, or other antidiabetic medications. Models were adjusted for day of the week, year, average temperature and relative humidity, gender, age, ethnicity, socioeconomic status, hypertension, and the purchase of antidiabetic medications 3 months before the test. IQR = interquartile range; ^∗^*P* < 0.05.

To evaluate possible interactions with the type of treatment among DM patients, we compared the percent change in SG levels among DM patients treated only with Metformin, insulin, or other oral medications, and patients that are not treated with antidiabetic medications. Weaker associations with NO_2_ were observed among untreated patients (1.16% increase in SG, 95% CI: 0.90–1.42%) and among patients treated with Metformin (0.56% increase in SG, 95% CI: 0.18–0.95%), compared to patients treated with Insulin (1.81% increase in SG, 95% CI: 0.26–3.38%) or other antidiabetic medications (2.12% increase in SG, 95% CI: 0.82–3.44%). No significant associations with SO_2_ were found in any of the sub groups (Figure 2).

Comparing the lowest and highest quantiles of coefficient of variance (CV) values, we found stronger association with NO_2_ among patients presented high variability in SG (1.53% increase in SG, 95% CI 0.42–2.65%), compared to the lowest quantile of SG variability (0.79% increase in SG, 95% CI: 0.34–1.25%). No significant associations were observed with SO_2_.

No effect modification was found in analyses stratified by age or ethnicity.

Approximately 4% (4756 subjects) of the study population had available HbA1c tests within the study period (a total of 5715 tests). HbA1c level ranged between 4% and 20.3%, with a median value of 6.7%. No associations were found between the different pollutants and HbA1c levels.

### Sensitivity Analysis

As a sensitivity analysis, we repeated the analyses using 2 methods of SES imputation: by assigning the most frequent SES level (low) and the highest SES level. The results were consistent with the main results and showed no difference in the inference.

## DISCUSSION

In this population-based study of 131,882 subjects with over 1 million glucose tests, we were able to detect associations of AP exposure and SG levels. We found positive associations between SG levels and NO_2_ and SO_2_, among subjects with normal glucose levels, IFG and diabetes. The association with NO_2_ was especially pronounced among DM patients; however, Metformin treatment ameliorated the negative effect of the pollutant.

### Short-Term Exposure

In the last few decades, studies have provided findings linking environmental exposures to IR, SG levels, and metabolic diseases.^2,20,21^ Yet, the evidence regarding the association of AP and glucose metabolism are sparse.^1,5,9,10,12,21^

Kim et al found positive associations of PM_10_, O_3_, and NO_2_ with IR. The authors contributed the lack of association found with SO_2_ to the different sources of the pollutants: while PM_10_, O_3_, and NO_2_ are emitted mostly from traffic sources, SO_2_ is known to be a combustion product in industries.^9^ We found positive associations between SG levels and NO_2_ and SO_2_, 72 and 24 hours concentrations before the test. In Southern Israel, other than natural dust and traffic as AP sources, another potential source is the emissions from an industrial site located approximately 15 km from the largest city in the area.

In accordance with our findings, a recent meta-analysis found a pooled relative risk of 1.08 (95% CI: 1.00, 1.17) for type 2 diabetes per 10 μg/m^3^ increase in NO_2_.^3^ Since the majority of NO_2_ emissions originate in traffic, the lack of significant associations reported in other studies^22,23^ may be due to low pollution levels and lower effect sizes.^10^

### Susceptible Populations

Previously, the majority of the epidemiological studies investigating AP effect on glucose homeostasis have focused on diabetic patients, as a group with particularly high susceptibility to AP-triggered cardiovascular events.^25–28^ Supporting the hypothesis of higher vulnerability of patients with DM, our study showed higher associations with both NO_2_ and SO_2_.

The association with NO_2_ was less pronounced among DM patients who presented low variability in SG results as well as among those treated with Metformin. The same inflammatory mechanism of AP associated with cardiovascular damage, is also believed to be involved in the promotion of IR and Type 2 diabetes.^22,29^ In the present study, we have shown that among patients with diabetes, those receiving Metformin treatment were resistant to the AP effect on glucose levels. Insulin sensitizers (eg, Metformin) suppress proinflammatory genes, therefore may enhance the antiinflammatory response occurring in the presence of AP exposure. Rioux and colleagues^30^ found lower c-reactive protein (CRP) levels among DM patients residing in proximity to main roads and treated with oral hypoglycemic medications versus nontreated, supporting the hypothesis of an antiinflammatory downregulation process.

### Health Implications

Assessing the results of our study, the main question that can be asked by the clinicians is: what is the health effect of these relatively numerically small observed associations between the air pollutants and glucose levels? The answer is 2-fold: the effect is clinically significant both on a population and individual levels. Given the broad extent of exposed population and the continuous nature of exposure, even small adverse associations represent a public health concern and may have implications on public health policies.^20^ Because the whole population is exposed, even small effects can be translated into substantial attributable adverse health outcomes.^31^

Another aspect that should be considered on an individual level is the chronic exposure effect. As short-term studies describe only part of the air-pollution-related adverse outcomes,^31^ while the cumulative lifetime risk is probably larger than the usually assessed acute risk. The cumulative adverse health effects are related to a combination of exposure intensity and duration. Therefore, even numerically small acute effects, such as observed in our study, can be translated into a profound detrimental clinical effect over longer period.

Glucose levels increasing due to the AP both within and above normal range can contribute to the development of vascular morbidity. As reported in the findings of the Honolulu Heart Study, the risk of CHD increases continuously as glucose levels increases,^32^ emphasizing the importance of glycemic control and glucose reduction, even in small amount and within the normal range. Furthermore, higher glucose levels were reported to be associated with cardiovascular disease, even within the range of normal glucose levels.^33^ That said, the AP related increases in glucose found in our study were relatively small. In order to establish cardiovascular risk the effect of lifetime chronic exposure should be estimated.

## LIMITATIONS

Our study had a number of limitations. First, AP data for our study period were available only from 1 monitoring site, located in the center of the largest city in the region. To reduce exposure measurement error we excluded subjects residing farther than 20 km from the monitoring site. Therefore, the rural and suburban population is underrepresented in our study. In addition, using this method, we were unable to estimate the variations in AP in rural versus urban locations. Second, the use of medications and laboratory results for DM definition might have resulted in misclassification, as well. Both limitations might have decreased the magnitude of the associations estimated in the study.

In addition, data of potential confounders such as BMI and smoking status were not available in this study, which precluded a more detailed adjustment of the study finding. SES level was assigned based on the SES of the population residing in the close proximity to the subjects’ primary clinic, but we did not have data regarding the exact estimated SES of each subject, therefore residual confounding by SES might still be present. Lastly, given the small amount of available HbA1c tests in our study, the analysis might not had the power required to detect relatively small differences and long-term associations.

## CONCLUSION

In summary, in this population-based study, we found small but consistent increases in SG associated with short-term exposure to NO_2_ and SO_2_; especially pronounced among patients with DM who were not treated with Metformin. Given the chronic lifetime exposure to AP and the broad extent of exposed population, even small associations such as those found in our study may have profound public and individual health implications.

## UNCITED REFERENCES

^19,24^

## References

[1] Sun Q Yue P Deiuliis JA Ambient air pollution exaggerates adipose inflammation and insulin resistance in a mouse model of diet-induced obesity. *Circulation* 2009; 119:538–546. 19153269 10.1161/CIRCULATIONAHA.108.799015 PMC3845676

[2] Pearson JF Bachireddy C Shyamprasad S Association between fine particulate matter and diabetes prevalence in the U.S. *Diabetes Care* 2010; 33:2196–2201. 20628090 10.2337/dc10-0698 PMC2945160

[3] Eze CI Hemkens GL Bucher CH Association between ambient air pollution and diabetes mellitus in Europe and North America: systematic review and meta-analysis. *Environ Health Perspect* 2015; 52:258–262. 10.1289/ehp.1307823 PMC4421762 25625876

[4] Andersen ZJ Raaschou-Nielsen O Ketzel M Diabetes incidence and long-term exposure to air pollution: a cohort study. *Diabetes Care* 2012; 35:92–98. 22074722 10.2337/dc11-1155 PMC3241311

[5] Chuang K-J Yan Y-H Cheng T-J Effect of air pollution on blood pressure, blood lipids, and blood sugar: a population-based approach. *J Occup Environ Med* 2010; 52:258–262. 20190657 10.1097/JOM.0b013e3181ceff7a

[6] Kim SY O’Neill MS Lee JT Air pollution, socioeconomic position, and emergency hospital visits for asthma in Seoul, Korea. *Int Arch Occup Environ Health* 2007; 80:701–710. 17357797 10.1007/s00420-007-0182-3

[7] Fleisch AF Gold DR Rifas-Shiman SL Abnormal glucose tolerance during pregnancy: the project Viva cohort. *Environ Health Perspect* 2014; 122:378–383. 24508979 10.1289/ehp.1307065 PMC3984217

[8] Park SK Wang W Ambient air pollution and type 2 diabetes mellitus: a systematic review of epidemiologic research. *Curr Environ Health Rep* 2014; 1:275–286. 25170433 10.1007/s40572-014-0017-9 PMC4142518

[9] Kim JH Hong Y-C GSTM1, GSTT1, and GSTP1 polymorphisms and associations between air pollutants and markers of insulin resistance in elderly Koreans. *Environ Health Perspect* 2012; 120:1378–1384. 22732554 10.1289/ehp.1104406 PMC3491923

[10] Teichert T Vossoughi M Vierkötter A Association between traffic-related air pollution, subclinical inflammation and impaired glucose metabolism: results from the SALIA study. *PLoS ONE* 2013; 8:e83042 doi: 10.1371/journal.pone.0083042. 24340078 10.1371/journal.pone.0083042 PMC3858363

[11] Thiering E Cyrys J Kratzsch J Long-term exposure to traffic-related air pollution and insulin resistance in children: results from the GINIplus and LISAplus birth cohorts. *Diabetologia* 2013; 56:1696–1704. 23666166 10.1007/s00125-013-2925-x PMC3699704

[12] Xu X Liu C Xu Z Long-term exposure to ambient fine particulate pollution induces insulin resistance and mitochondrial alteration in adipose tissue. *Toxicol Sci* 2011; 124:88–98. 21873646 10.1093/toxsci/kfr211 PMC3196653

[13] Zheng Z Xu X Zhang X Exposure to ambient particulate matter induces a NASH-like phenotype and impairs hepatic glucose metabolism in an animal model. *J Hepatol* 2013; 58:148–154. 22902548 10.1016/j.jhep.2012.08.009 PMC3527686

[14] Brook RD Xu X Bard RL Reduced metabolic insulin sensitivity following sub-acute exposures to low levels of ambient fine particulate matter air pollution. *Sci Total Environ* 2013; 448:66–71. 22901427 10.1016/j.scitotenv.2012.07.034 PMC4391076

[15] Chen J-C Schwartz J Metabolic syndrome and inflammatory responses to long-term particulate air pollutants. *Environ Health Perspect* 2008; 116:612–617. 18470293 10.1289/ehp.10565 PMC2367655

[16] Peled R Tal A Pliskin JS A computerized surveillance system for the quality of care in childhood asthma. *J Healthc Qual* 2005; 27:28–33. 17514847 10.1111/j.1945-1474.2005.tb00574.x

[17] Central Bureau of Statistics (CBS). Characterization and classification of geographical units by the socio-economic level of the population. 2008 http://www.cbs.gov.il/webpub/pub/text_page_eng.html?publ=100&CYear=2008&CMonth=1 [Accessed December 22, 2014].

[18] American Diabetes Association Diagnosis and classification of diabetes mellitus. *Diabetes Care* 2014; 37 (Suppl. 1):S81–S90. 24357215 10.2337/dc14-S081

[19] Ganor E Stupp A Alpert P A method to determine the effect of mineral dust aerosols on air quality. *Atmos Environ* 2009; 43:5463–5468.

[20] Rajagopalan S Brook RD Air pollution and type 2 diabetes: mechanistic insights. *Diabetes* 2012; 61:3037–3045. 23172950 10.2337/db12-0190 PMC3501850

[21] Chuang K-J Yan Y-H Chiu S-Y Long-term air pollution exposure and risk factors for cardiovascular diseases among the elderly in Taiwan. *Occup Environ Med* 2011; 68:64–68. 20833756 10.1136/oem.2009.052704

[22] Brook RD Jerrett M Brook JR The relationship between diabetes mellitus and traffic-related air pollution. *J Occup Environ Med* 2008; 50:32–38. 18188079 10.1097/JOM.0b013e31815dba70

[23] Dijkema MB Mallant SF Gehring U Long-term exposure to traffic-related air pollution and type 2 diabetes prevalence in a cross-sectional screening-study in the Netherlands. *Environ Health* 2011; 10:10–1186.doi: 10.1186/1476-069X-10-76. 21888674 10.1186/1476-069X-10-76 PMC3200985

[24] Tamayo T Rathmann W Krämer U Is particle pollution in outdoor air associated with metabolic control in type 2 diabetes? *PloS ONE* 2014; 9:e91639. 24619127 10.1371/journal.pone.0091639 PMC3950252

[25] O’Neill MS Veves A Zanobetti A Diabetes enhances vulnerability to particulate air pollution—associated impairment in vascular reactivity and endothelial function. *Circulation* 2005; 111:2913–2920. 15927967 10.1161/CIRCULATIONAHA.104.517110

[26] Jarczok MN Li J Mauss D Heart rate variability is associated with glycemic status after controlling for components of the metabolic syndrome. *Int J Cardiol* 2013; 167:855–861. 22386703 10.1016/j.ijcard.2012.02.002

[27] Schneider A Neas LM Graff DW Association of cardiac and vascular changes with ambient PM2.5 in diabetic individuals. *Part Fibre Toxicol* 2010; 7:14 doi: 10.1186/1743-8977-7-14. 20525188 10.1186/1743-8977-7-14 PMC2896918

[28] Zanobetti A Schwartz J Cardiovascular damage by airborne particles: are diabetics more susceptible? *Epidemiology (Cambridge, Mass)* 2002; 13:588–592. 10.1097/00001648-200209000-00016 12192230

[29] Wellen KE Hotamisligil GS Inflammation, stress, and diabetes. *J Clin Invest* 2005; 115:1111–1119. 15864338 10.1172/JCI25102 PMC1087185

[30] Rioux CL Tucker KL Brugge D Traffic exposure in a population with high prevalence type 2 diabetes—do medications influence concentrations of C-reactive protein? *Environ Pollut (Barking, Essex: 1987)* 2011; 159:2051–2060. 10.1016/j.envpol.2010.12.025 PMC3412137 21292365

[31] Kunzli N Kaiser R Medina S Public-health impact of outdoor and traffic-related air pollution: a European assessment. *Lancet* 2000; 356:795–801. 11022926 10.1016/S0140-6736(00)02653-2

[32] Scheidtnave C Barrettconnor E Wingard DL Sex-differences in fasting glycemia as a risk factor for ischemic-heart-disease death. *Am J Epidemiol* 1991; 133:565–576. 2006643 10.1093/oxfordjournals.aje.a115928

[33] Gerstein HC Glucose: a continuous risk factor for cardiovascular disease. *Diabet Med* 1997; 14:S25–S31. 9272610 10.1002/(sici)1096-9136(199708)14:3+<s25::aid-dia441>3.3.co;2-t

